# A programmable system to methylate and demethylate *N*^6^-methyladenosine (m^6^A) on specific RNA transcripts in mammalian cells

**DOI:** 10.1016/j.jbc.2022.102525

**Published:** 2022-09-23

**Authors:** Chen Chang, Gang Ma, Edwin Cheung, Andrew P. Hutchins

**Affiliations:** 1 Department of Biology, School of Life Sciences, Southern University of Science and Technology, Shenzhen, China; 2 Cancer Centre, Faculty of Health Sciences, University of Macau, Taipa, China; 3 Shenzhen Key Laboratory of Gene Regulation and Systems Biology, School of Life Sciences, Southern University of Science and Technology, Shenzhen, China

**Keywords:** CRISPR/Cas13a, m^6^A, epitranscriptomics, X-chromosome inactivation, Cas, CRISPR-associated protein, cDNA, complementary DNA, crRNA, CRISPR RNA, ESC, embryonic stem cell, HEK293T, human embryonic kidney 293T cell line, IP, immunoprecipitation, IRES, internal ribosome entry site, lncRNA, long noncoding RNA, m^6^A, *N*^6^-methyladenosine, MeRIP–qPCR, m^6^A RNA immunoprecipitation–qPCR, mESC, mouse embryonic stem cell, MTD, methyltransferase domain, qPCR, quantitative PCR, *XIST*, X-inactive specific transcript

## Abstract

RNA *N*^6^-methyladenosine (m^6^A) is the most abundant internal mRNA modification and forms part of an epitranscriptomic system that modulates RNA function. m^6^A is reversibly catalyzed by specific enzymes, and those modifications can be recognized by RNA-binding proteins that in turn regulate biological processes. Although there are many reports demonstrating m^6^A participation in critical biological functions, this exploration has mainly been conducted through the global KO or knockdown of the writers, erasers, or readers of m^6^A. Consequently, there is a lack of information about the role of m^6^A on single transcripts in biological processes, posing a challenge in understanding the biological functions of m^6^A. Here, we demonstrate a CRISPR/dCas13a-based RNA m^6^A editors, which can target RNAs using a single or multiple CRISPR RNA array to methylate or demethylate m^6^A in human 293T cells and mouse embryonic stem cells. We systematically assay its capabilities to enable the targeted rewriting of m^6^A dynamics, including modulation of circular RNA translation and transcript half-life. Finally, we use the system to specifically modulate m^6^A levels on the noncoding *XIST* (X-inactive specific transcript) to modulate X chromosome silencing and activation. The editors described here can be used to explore the roles of m^6^A in biological processes.

Analogous to the DNA epigenetic system, RNA is also chemically modified to impart epitranscriptomic information. *N*^6^-methyladenosine (m^6^A) is the most abundant internal RNA chemical modification in higher eukaryotes and marks all classes of RNA, including coding and noncoding transcripts (1, 2, 3). In a typical cell type in mammals, several thousand transcripts are extensively marked by m^6^A (4, 5), generally in the 3′UTR in coding transcripts (4), but anywhere in noncoding transcripts (6). Increasingly, m^6^A has been implicated in a diverse range of cellular processes, centered on RNA metabolism and processing, including RNA stability, alternative splicing, nuclear export, retrotransposon silencing, and protein translation (7, 8, 9, 10, 11, 12, 13, 14, 15, 16, 17, 18). In addition, m^6^A has also been recognized to play a role in several biological processes and diseases, including embryonic stem cell differentiation (12, 19), oncogenesis (15, 20, 21, 22, 23, 24), neurogenesis (25, 26), and X-chromosome inactivation (7, 27, 28, 29, 30), amongst many others (1, 21, 31, 32). However, these mechanisms have been largely explored through the global KO or knockdown of m^6^A effectors, such as writers (METTL3/METTL14/WTAP/RBM15/15B), erasers (FTO/ALKBH5), or the readers (YTH protein family) (12, 15, 17, 23, 31, 33). Considering m^6^A marks several thousand transcripts in a typical cell type, the scope for pleiotropic effects from these knockdowns/KOs is large. Hence, to reveal the specific functions of m^6^A on individual transcripts, there is a need to specifically modulate m^6^A on single or multiple transcripts.

The CRISPR systems from bacteria or archaea utilize CRISPR RNAs (crRNAs) to guide CRISPR-associated proteins (Cas) specifically cleave invading DNA- and RNA-based pathogens (34, 35, 36, 37). This bacterial immune system has been repurposed to create a powerful suite of genome and epigenome modification tools (38, 39, 40, 41, 42, 43, 44, 45, 46). Whilst many Cas proteins such as Cas9 target DNA, there are also Cas proteins that target RNA (40, 45, 46). LwaCas13a contains two RNase domains and can degrade RNA guided by a crRNA sequence (47, 48, 49, 50, 51). A catalytic-dead Cas13a was generated (dCas13a), which when guided by a crRNA, can function as an RNA sequence–specific binding protein that can deliver dCas13a-fused proteins to specific RNAs (47, 52, 53). Thus, catalytic-dead dCas13a, fused to RNA-modifying enzymes, cotransfected with specific crRNAs can potentially modulate information on single transcripts. To date, several m^6^A-editing systems have been characterized, dCas13b-METTL3 (54), dCas9-METTL3-METTL14 (55), dCas13b-ALKBH5 (56), and dCas13a-ALKBH5 (57), dCas13b/SunTag (58), dCasRx-METTL3/ALKBH5 (59), and a photoactivatable system (60). The advantages and disadvantages of these systems were recently reviewed (61).

Here, we generated dCas13a fused with full-length METTL3 or the core m^6^A methyltransferase domain (MTD) containing the methylation domain and two core zinc finger domains of METTL3 (62, 63, 64, 65) or with the m^6^A demethylase FTO (66, 67). The dCas13a fusions are available in vectors compatible with liposomal transfection or lentiviral vectors suitable for hard-to-transfect cells. The system works in human and mouse cells. We show that our CRISPR/dCas13a-associated RNA m^6^A-editing system can be specifically navigated by crRNAs to modulate m^6^A on individual coding or noncoding RNAs. We demonstrate the efficacy of this system to modulate circular RNA translational efficiency and RNA transcript half-life. In addition, by taking advantage of the intrinsic capability for Cas13a to mature pre-crRNA, we show how the dCas13a fusions can process a programmable crRNA array containing multiple crRNAs to target several transcripts simultaneously for m^6^A editing. Finally, we utilize this system to edit m^6^A on the long noncoding RNA (lncRNA) *XIST* (X-inactive specific transcript), resulting in the reactivation of *XIST*-repressed genes, or active silencing in *METTL3* knockdown cells. This work describes a series of tools that can edit m^6^A in multiple mammalian cellular contexts on specific RNAs to explore the effect of m^6^A on transcript activity.

## Results

### Construction of dCas13a-associated m^6^A-editing systems in mammalian cells

m^6^A RNA methylation is catalyzed by several protein complexes containing multiple protein subunits, for example, METTL3, METTL14, WTAP, and so on (32, 62, 63, 68, 69, 70, 71). However, amongst these proteins, METTL3 is the catalytic enzyme (63, 65). m^6^A demethylation can be accomplished by a single catalytic enzyme (62, 67, 69, 71), such as FTO or ALKBH5. Hence, we selected human METTL3 full-length protein, or a truncated methylation domain (MTD), containing the methylation domain and two core zinc finger domains, as m^6^A writers, and FTO as an m^6^A eraser.

**Figure 1:**
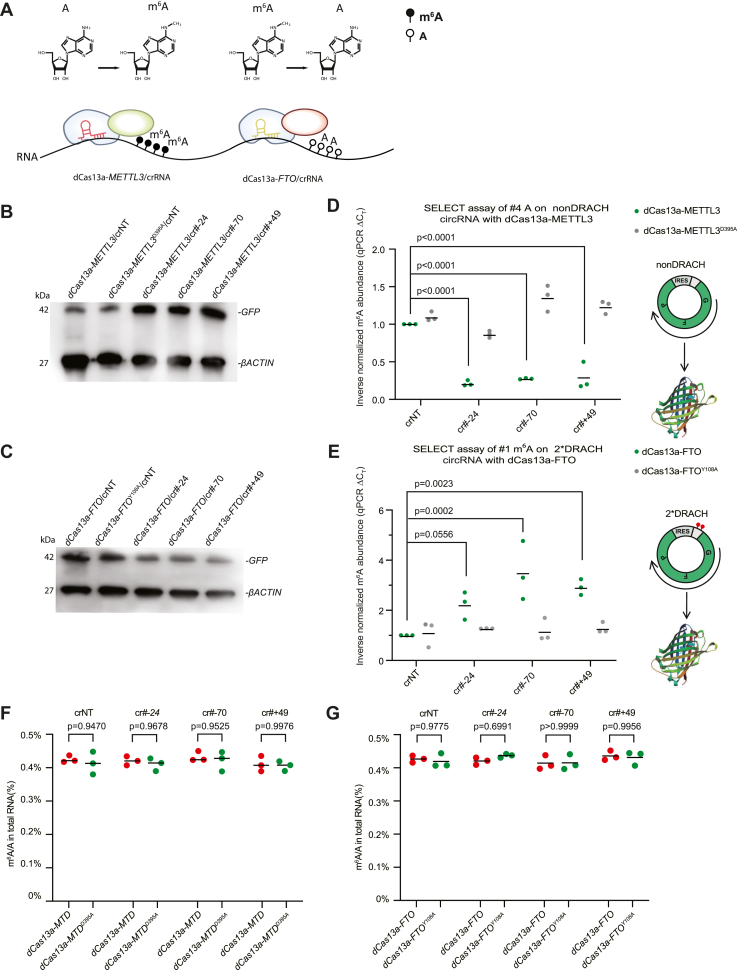
**A programmable m**^**6**^**A methylation and demethylation system.***A*, schematic of the m^6^A-editing systems. *B*, Western blot of GFP or βACTIN from 293T cells transfected with crNT (nontargeting crRNA) or crRNAs targeting specific sites around the ATG codon of the circular nonDRACH-containing plasmid (Fig. S2*D*), and dCas13a-METTL3, or the catalytic-dead dCas13a-METTL3^D395A^. This experiment was repeated three times with similar results. Molecular weight markers (in kilodalton) are indicated on the *left side* for this and all subsequent Western blots. *C*, Western blot of GFP or βACTIN from 293T cells transfected with crNT or crRNAs targeting specific sites around the ATG codon of the 2-DRACH-containing circular plasmid (Fig. S2*D*) and dCas13a-FTO or the catalytic-dead dCas13a-FTO^Y108A^. This experiment was repeated three times with similar results. *D*, SELECT assay of m^6^A level on nonDRACH circGFP at position 22 (Fig. S2*A*), in cells transfected with dCas13a-METTL3, the catalytic-null catalytic-dead dCas13a-METTL3^D395A^, and crRNAs targeting the indicated base pairs relative to the GFPs ATG. *Y*-axis indicates inverse normalized m^6^A abundance normalized to dCas13a-METTL3/crNT sample. Significance is from a two-tailed unpaired Student’s *t* test, for this and all statistical tests unless otherwise stated. Dots indicate the mean for each biological replicate, and the bar is the mean of all biological replicates, n = 3 biological replicates with three technical replicates each. *E*, SELECT assay of m^6^A level on 2DRACH circGFP at position 4 (Fig. S2*A*), in cells transfected with dCas13a-FTO, the catalytic-null catalytic-dead dCas13a-FTO^Y108A^, and crRNAs targeting the indicated base pairs relative to the GFPs ATG. *Y*-axis indicates inversed normalized m^6^A abundance normalized to dCas13a-FTO/crNT sample. *Dots* indicate the mean for each biological replicate, and the *bar* is the mean of all biological replicates, n = 3 biological replicates with three technical replicates each. *F*, whole transcriptome m^6^A measurement for RNA m^6^A level in 293T cells transfected with the indicated crRNAs and dCas13a-METTL3, catalytic-dead dCas13a-METTL3^D395A^. *Dots* indicate the mean for each biological replicate, and the *bar* is the mean of all biological replicates, n = 3 biological replicates with three technical replicates each. *p* Value is from a one-way ANOVA. *G*, whole transcriptome m^6^A measurement for RNA m^6^A level in293T cells transfected with the indicated crRNAs and dCas13a-FTO or catalytic-dead dCas13a-FTO^Y108A^. *Dots* indicate the mean for each biological replicate, and the *bar* is the mean of all biological replicates, n = 3 biological replicates with three technical replicates each. *p* Value is from a one-way ANOVA. m^6^A, *N*^6^-methyladenosine.

To construct an m^6^A-editing system, we fused the catalytically inactivated LwaCas13a (dCas13a) (47, 48) protein with METTL3/MTD or FTO (Figs. 1*A* and S1*A*). As controls, we constructed catalytic-dead mutants of METTL3^D395A^ (63, 65) and FTO^Y108A^ (67) fused to dCas13a (Fig. S1*A*). All these fusion systems contain an N-terminal dCas13a, followed by a 13 amino acid GGS linker, a hemagglutinin tag, three nuclear localization signals, and a P2A (self-cleaving peptide sequence) fused with mCherry to report transfection efficiency (Fig. S1*A*). The crRNA is contained on a second U6-promoter–containing vector, which is cotransfected along with the fusion protein (Fig. S1*A*). After transfection of the editing systems into 293T cells, mCherry fluorescence was detected and transfection efficiency was estimated as 62% (Fig. S1, *B*–*E*). Importantly, transfection of dCas13a-METTL3 or dCas13a-FTO did neither substantially alter the cell cycle nor increase the proportion of dead cells (Fig. S1, *F*–*H*), indicating the transgenes were not inducing cellular toxicity. Together, these vectors make up an m^6^A-editing system that can be targeted by the crRNA to a specific transcript.

### Manipulating m^6^A on exogenous circular RNAs modulates their translation

To explore the activity of the dCas13a fusions, we initially constructed a reporter system to indirectly measure the m^6^A levels of a specific RNA. To this end, we exploited the ability of m^6^A to promote protein translation efficiency from circular RNAs in human cells (72, 73, 74). To determine if our system can modify specific m^6^A sites on RNA, we generated a circular RNA expression system to serve as an indirect readout of m^6^A (Fig. S2*A*) (72, 74). We generated an artificial circular RNA expression system where the coding sequence of GFP was separated linearly into two parts between an IRES (internal ribosome entry site) to form an “FP-IRES-(ATG)G” linear sequence that would back-splice to form a complete circular IRES-GFP coding sequence (Fig. S2*A*). Based on several reports (4, 5, 75), the METTL3–METTL14–WTAP complex prefers to methylate the A in a DRACH consensus motif, where D donates A, G, or U, R donates A or G, and H donates A, C, or U nucleotides (Fig. S2*B*). Hence, we generated a series of reporters that include 2, 1, and 0 GGACU sequences or no A nucleotides (Fig. S2*C*). In the endogenous METTL3–METTL14 complex, METTL14 targets METTL3 to DRACH motifs (4, 5, 75); however, as our system does not use METTL14, the requirement for DRACH targeting is relaxed, and potentially any available A nucleotide is a target. Consequently, we used the no DRACH (low levels of translation) and the 2-DRACH (high levels of translation) circular reporters as an indirect readout for changes in m^6^A levels (Fig. S2, *B* and *C*).

Several sites were selected as targets for a crRNA, including locations just outside the IRES region, and before or after the start codon (defined as position 1) of the GFP. crRNAs were constructed targeting specific sequences (Fig. S2*D* and Table S1). We then cotransfected a crRNA with dCas13a-METTL3 with the no-DRACH reporter. In a separate experiment, we used dCas13a-FTO cotransfected with the 2-DRACH plasmid. We assayed the indirect impact of m^6^A on protein translation by measuring the level of GFP in a Western blot. Western blot showed that, compared with nontargeting crRNA, crRNAs targeting the circular RNA resulted in improved GFP translation from the dCas13a-METTL3 transfected cells and impaired translation when using the dCas13a-FTO system (Figs. 1, *B* and *C* and S3, *A* and *B*). Interestingly, GFP expression was affected even when the crRNA was up to 107 nucleotides 5′ of the ATG, in the case of dCas13a-METTL3 (Fig. S3*C*) or up to 95 nucleotides 5′ of the ATG, in the case of dCas13a-FTO (Fig. S3*D*). We then performed an assay based on competitive elongation and ligation-based quantitative PCR (qPCR) to directly measure the level of m^6^A at a specific nucleotide (“SELECT” assay) (76). In the SELECT qPCR-based assay, the presence of an m^6^A impairs the production of the complementary DNA (cDNA) product. Increased m^6^A manifests as a decreased cycle threshold, whereas decreased m^6^A increases the cycle threshold (76). SELECT assay showed that dCas13a-METTL3 with specific crRNAs resulted in upregulation of m^6^A, which was not present when using the catalytic mutant form of METTL3^D395A^ (Fig. 1*D*). Conversely, dCas13a-FTO decreased m^6^A levels, whereas dCas13a-FTO^Y108A^ catalytic mutant did not change m^6^A levels (Fig. 1*E*). One concern is that the overexpression of dCas13a-METTL3/FTO may act as a dominant positive and nonspecifically methylate RNA. Assay for the transcriptome-wide level of m^6^A indicated that the dCas13a fusions did not significantly change the transcriptome level of m^6^A (Fig. 1, *F* and *G*). We also confirmed that the dCas13a-METTL3/FTO constructs did not alter endogenous METTL3 or FTO protein levels (Fig. S3, *E* and *F*), and the dCas fusions were only expressed at around 20% of the level of the endogenous protein (Fig. S3, *G* and *H*). Finally, there was no effect on endogenous METTL3 when dCas13a-FTO was expressed nor on the inverse (Fig. S3, *I* and *J*).

### dCas13a-METTL3, dCas13a-MTD, and dCas13a-FTO fusions can modulate m^6^A on endogenous transcripts and alter their half-life in human embryonic kidney 293T cells

**Figure 2:**
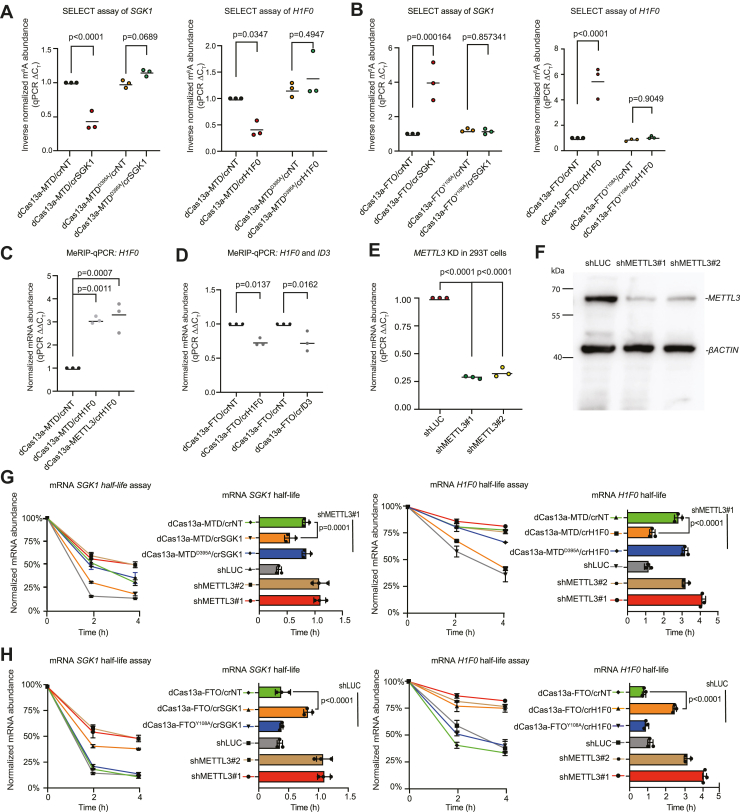
**m**^**6**^**A editors can alter the half-life of specific endogenous transcripts.***A*, SELECT assay for m^6^A level on *SGK1* and *H1F0*, with dCas13a-MTD and catalytic-null and crRNA targeting *SGK1* or *H1F0*. *Y*-axis indicates inverse normalized m^6^A abundance normalized to dCas13a-MTD/crNT sample. *Dots* indicate the mean for each biological replicate, and the *bar* is the mean of all biological replicates, n = 3 biological replicates with three technical replicates each. *B*, SELECT assay for m^6^A level on *SGK1* and *H1F0*, with dCas13a-FTO and catalytic-null and crRNA targeting *SGK1* or *H1F0*. *Y*-axis indicates inverse normalized m^6^A abundance normalized to dCas13a-FTO/crNT sample. *Dots* indicate the mean for each biological replicate, and the *bar* is the mean of all biological replicates, n = 3 biological replicates with three technical replicates each. *C*, MeRIP–qPCR assay in 293T cells with dCas13a-MTD cotransfected with crRNA against *H1F0*. Data were normalized to the dCas13a-MTD/crNT sample and against the input. *Dots* indicate the mean for each biological replicate, and the *bar* is the mean of all biological replicates, n = 3 biological replicates with three technical replicates each. *D*, MeRIP–qPCR assay in 293T cells with dCas13a-FTO cotransfected with crRNAs against *H1F0* or *ID3*. Data were normalized to the dCas13a-FTO/crNT sample and against the input. *Dots* indicate the mean for each biological replicate, and the *bar* is the mean of all biological replicates, n = 3 biological replicates with three technical replicates each. *E*, RT–qPCR of *METTL3* in 293T cells transfected with shLUC (control shRNA against luciferase) or shMETTL3#1 and shMETTL3#2. Data were normalized to the shLUC sample and *ACTB*. *F*, Western blot of METTL3 and βACTIN in 293T cells transfected with shLUC or shMETTL3#1 and shMETTL3#2. *G*, *SGK1* and *H1F0* half-life assay, with dCas13a-MTD or the catalytic-null cotransfected with control crNT or crRNA against *SGK1* or *H1F0*. Data are represented as the SEM, n = 3 biological replicates with three technical replicates each. 293T cells transfected with an shLUC (luciferase control), shMETTL3#1, or shMETTL3#2 are shown for comparison. Data were normalized to their respective 0 h time points. The *right-hand bar charts* show the calculated transcript half-lives. *H, SGK1* and *H1F0* half-life assay, with dCas13a-FTO or the catalytic-null cotransfected with control crNT or crRNA against *SGK1* or *H1F0*. Data are represented as the SEM, n = 3 biological replicates with three technical replicates each. 293T cells transfected with an shLUC (luciferase control) shMETTL3#1 or shMETTL3#2 are shown for comparison. Data were normalized to their respective 0 h time points. The *right-hand bar charts* show the calculated transcript half-lives. crRNA, CRISPR RNA; m^6^A, *N*^6^-methyladenosine; MeRIP–qPCR, m^6^A RNA immunoprecipitation–qPCR; MTD, methyltransferase domain; qPCR, quantitative PCR.

We next set out to use the dCas13a fusions on endogenous transcripts. To verify whether the dCas13a fusions can edit m^6^A on endogenous transcripts, we chose several candidate RNAs expressed in human embryonic kidney 293T (HEK293T) cells, including mRNAs (*H1F0*, *SGK1*, and *ID3*) and an lncRNA (*MALAT1*). Reanalysis of m^6^A sequencing data in 293T cells indicated these transcripts are expressed and marked by m^6^A (Fig. S4, *A*–*D*) (4, 9, 33, 77). We next transfected cells with the dCas13a fusions and crRNAs. SELECT assay indicated that m^6^A levels of *H1F0* and *SGK1* were significantly increased in the dCas13a-MTD but were unaltered with the nontargeting crRNA or with the MTD catalytic mutant (Fig. 2*A*). dCas13a-FTO with the same crRNAs against *H1F0* and *SGK1* led to a significant decrease in m^6^A levels (Fig. 2*B*). m^6^A RNA immunoprecipitation (IP)–qPCR (MeRIP–qPCR) supported the results from SELECT, and indicated dCas13a-MTD resulted in significantly increased m^6^A levels on *H1F0* transcripts (Fig. 2*C*), whereas dCas13a-FTO led to a significant decrease in m^6^A on *H1F0* or *ID3* transcripts when the respective crRNAs were cotransfected (Fig. 2*D*).

We next explored m^6^A on the lncRNA *MALAT1*. Using crRNAs specific to *MALAT1*, transfection of dCas13a-MTD significantly increased m^6^A levels, as measured by both SELECT and MeRIP–qPCR (Fig. S5, *A* and *B*), whereas dCas13a-FTO significantly reduced m^6^A levels (Fig. S5, *C* and *D*). Importantly, SELECT indicated that these changes in m^6^A levels were only achieved when using catalytically active dCas13a-MTD/METTL3/FTO, as the catalytic mutants did not affect m^6^A levels (Fig. S5, *A*–*D*). We confirmed that the dCas13a-MTD/FTO fusions were not acting as dominant positives, as there were no significant changes in the whole transcriptome level of m^6^A (Fig. S5, *E* and *F*). We also explored some off-target effects and used SELECT assay to show there was no off-target methylation/demethylation of *MALAT1* when a crRNA against *SGK1* of *H1F0* was cotransfected with dCas13a-MTD or dCas13a-FTO (Fig. S5, *G* and *H*). These data indicate that there is no general modulation of m^6^A on RNAs, although we cannot rule out off-target effects on other RNAs.

When m^6^A writers or readers knocked down or knocked out RNA, half-life has been reported to increase (9, 12, 33). However, these results have been demonstrated mainly in global KO or knockdowns of m^6^A effectors. We wondered if our system could be used to alter the half-life of specific transcripts by changing the level of m^6^A on target RNAs. First, we validated two shRNAs to knock down *METTL3* in 293T cells (9, 20). Both shRNAs efficiently depleted both the mRNA and protein levels of METTL3 (Fig. 2, *E* and *F*). There was no change in cell morphology or the number of cells dying (Fig. S5*I*). However, there was an increase in the percentage of G2/M phase cells in the *METTL3* knockdowns, suggesting METTL3 is involved in regulating cell cycle–related transcripts. Cells were treated with actinomycin D to block transcription, and RNA abundance was measured by qRT–PCR (9). For *METTL3* knockdown in 293T cells, we focused on two RNAs, *SGK1* and *H1F0*, as their half-life has been previously characterized as influenced by m^6^A levels (33). The results showed that dCas13a-MTD, in cells with *METTL3* knockdown, significantly decreased the stability of *SGK1* and *H1F0* and reduced their half-lives close to WT levels (Fig. 2*G*). Conversely, dCas13a-FTO, with crRNAs against *SGK1* or *H1F0*, significantly increased the respective mRNA half-lives in WT cells and brought them close to the corresponding transcript half-life in the *METTL3* knockdown cells (Fig. 2*H*). This demonstrates a direct effect of m^6^A on the half-life of specific transcripts.

### dCas13a-METTL3 and dCas13a-FTO fusions can alter mouse embryonic stem cell–specific RNA half-lives by modulating m^6^A on endogenous transcripts in mouse embryonic stem cells

**Figure 3:**
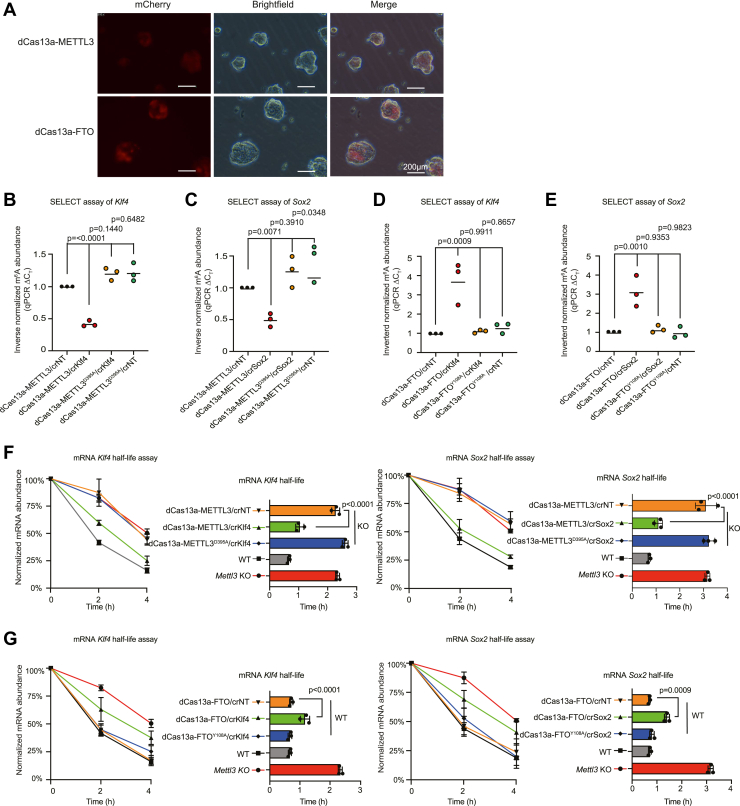
**m**^**6**^**A editors can alter the m**^**6**^**A level and half-life of specific transcripts in mESCs.***A*, fluorescence microscope images of mESCs transfected with dCas13a-METTL3 or dCas13a-FTO showing mCherry and brightfield views. The scale bar represents 200 μm. *B*, SELECT assay for m^6^A level on *Klf4*, with dCas13a-METTL3 or dCas13a-METTL3^D395A^ catalytic null with crRNA targeting *Klf4* or a control nontargeting (crNT) crRNA. *Y*-axis indicates inverse normalized m^6^A abundance normalized to dCas13a-METTL3/crNT sample. Data are represented as the SEM, n = 3 biological replicates with three technical replicates each. *C*, SELECT assay for m^6^A level on *Sox2*, with dCas13a-METTL3 or dCas13a-METTL3^D395A^ catalytic null with crRNA targeting *Sox2* or a control crNT crRNA. *Y*-axis indicates inverse normalized m^6^A abundance normalized to dCas13a-METTL3/crNT sample. Data are represented as the SEM, n = 3 biological replicates with three technical replicates each. *D*, SELECT assay for m^6^A level on *Klf4*, with dCas13a-FTO or dCas13a-FTO^Y108A^ catalytic null with crRNA targeting *Klf4* or a control crNT crRNA. *Y*-axis indicates inverse normalized m^6^A abundance normalized to dCas13a-FTO/crNT sample. Data are represented as the SEM, n = 3 biological replicates with three technical replicates each. *E*, SELECT assay for m^6^A level on *Sox2*, with dCas13a-FTO or dCas13a-FTO^Y108A^ catalytic null with crRNA targeting *Sox2* or a control crNT crRNA. *Y*-axis indicates inverse normalized m^6^A abundance normalized to dCas13a-FTO/crNT sample. Data are represented as the SEM, n = 3 biological replicates with three technical replicates each. *F, Klf4* and *Sox2* half-life assay, with dCas13a-METTL3 or its catalytic null cotransfected with a control crRNA or crRNA against *Klf4* or *Sox2* into *Mettl3* KO cells. Data from WT and KO *Mettl3* mESCs are shown for comparison. Data are represented as the SEM, n = 3 biological replicates with three technical replicates each. Data were normalized to their respective 0 h time points. The *right-hand bar charts* show the calculated transcript half-life. *G, Klf4* and *Sox2* half-life assay, with dCas13a-FTO or its catalytic null cotransfected with a control crRNA or crRNA against *Klf4* or *Sox2* into WT cells. Data from WT and KO *Mettl3* mESCs are shown for comparison. Data are represented as the SEM, n = 3 biological replicates with three technical replicates each. Data were normalized to their respective 0 h time points. The *right-hand bar charts* show the calculated transcript half-life. crRNA, CRISPR RNA; m^6^A, *N*^6^-methyladenosine; mESC, mouse embryonic stem cell.

Several research groups have demonstrated that m^6^A plays a critical role in regulating mouse embryonic stem cell (mESC) fate transitions (1, 12, 19, 32, 78). mESCs are resistant to transfection with liposomal techniques; hence, we generated dCas13a fusions in combined crRNA lentiviral vectors (Fig. S6*A*). These vectors are one-component vectors containing both the fusion protein and the crRNA. The lentiviral vector is suitable for difficult-to-transfect cell lines, such as mouse and human embryonic stem cells (ESCs), and when transfected into mESCs, mCherry was detected (Fig. 3*A*). We utilized *Mettl3* KO mESCs grown under naïve “2iLIF” (medium including serum, PD98059, CHIR99021, and leukemia inhibitory factor) conditions (12) (Fig. S6*B*) and designed crRNAs targeting two RNAs expressed at high levels in mESCs that are marked by m^6^A: *Klf4* and *Sox2* (Fig. S6, *C* and *D*). We confirmed that KO of *Mettl3* led to increased RNA half-lives of *Klf4* and *Sox2* compared with WT cells (Fig. S6, *E* and *F*). SELECT assay showed that expression of dCas13a-METTL3 led to significantly increased m^6^A levels on *Klf4* and *Sox2* mRNAs when the corresponding crRNA was present (Fig. 3, *B* and *C*). This effect was lost if the dCas13a-METTL3^D395A^ catalytic mutant was used (Fig. 3, *B* and *C*). dCas13a-FTO, conversely, led to decreased m^6^A levels, and the dCas13a-FTO^Y108A^ catalytic-null FTO had no effect on m^6^A (Fig. 3, *D* and *E*). Next, we measured the effect of the dCas13a fusions on RNA half-lives. In *Mettl3* KO mESCs when dCas13a-METTL3 was transfected with the corresponding crRNA, the half-lives of *Klf4* and *Sox2* were significantly shorter compared with the *Mettl3* KO cells or crRNA control (crNT) or catalytic-null dCas13a-METTL3^D395A^ (Fig. 3*F*). Conversely, dCas13a-FTO mediated the opposite effect, and the half-lives of *Klf4* and *Sox2* significantly increased, although they did not reach the half-lives in the *Mettl3* KO mESCs, suggesting dCas13a-FTO does not completely demethylate the transcripts (Fig. 3*G*). These results show that the dCas13a-METTL3 and dCas13a-FTO lentiviral systems can modulate m^6^A levels and transcript half-lives in mESCs.

### X chromosome-suppressed genes can be reactivated or resilenced by modulating m^6^A on *XIST*

In somatic female cells, only one X chromosome is active. The inactivated X chromosome is silenced through the activity of the noncoding RNA *XIST*, which coats one of the two chromosomes and recruits polycomb proteins and other epigenetic repressors (79, 80, 81, 82, 83, 84). The *XIST* lncRNA is heavily m^6^A methylated, and when m^6^A readers were knocked down, X chromosome genes were reactivated (7, 27, 28, 32). These results suggest that m^6^A plays a critical role in X chromosome inactivation. However, as m^6^A marks many thousands of transcripts, the role of m^6^A specifically on *XIST* and whether it is a determinant for X chromosome silencing is unclear.

**Figure 4:**
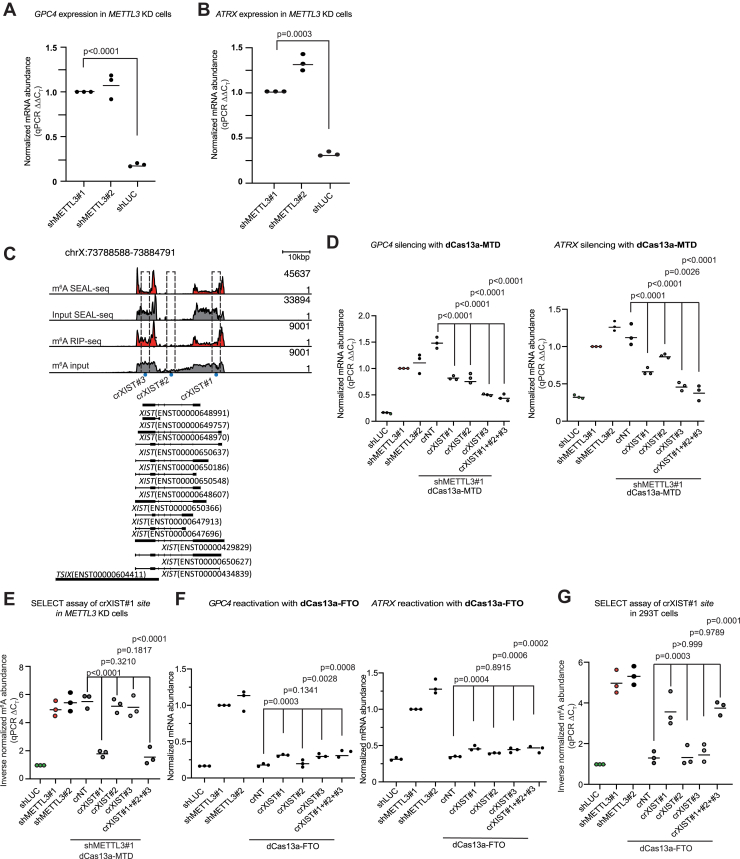
**m**^**6**^**A editors can modulate *XIST* function and reactivate X chromosome-inactivated genes**. *A*, RT–qPCR of the X chromosome gene *GPC4* when *METTL3* was knocked down in 293T cells. *Dots* indicate the mean for each biological replicate, and the *bar* is the mean of all biological replicates, n = 3 biological replicates with three technical replicates each. Data are normalized to the shLUC sample and *ACTB*. *B*, RT–qPCR of the X chromosome gene *ATRX* when *METTL3* was knocked down in 293T cells. *Dots* indicate the mean for each biological replicate, and the *bar* is the mean of all biological replicates, n = 3 biological replicates with three technical replicates each. Data are normalized to the shLUC sample and *ACTB*. *C*, genome view (hg38 genome assembly) of m^6^A RIP-Seq data in 293T cells at the *XIST* locus. The locations of the crRNAs are indicated with a *dotted box*. *Red* indicates m^6^A enrichment data, and *gray tracks* indicate the corresponding input data. Transcripts are from GENCODE, version 32. m^6^A abundance data are from GSE129979 (91) (*top two rows*) or GSE29714 (4) (*bottom two rows*). *D*, RT–qPCR of *GPC4* or *ATRX* when *METTL3* was knocked down, compared with cells cotransfected with dCas13a-MTD and crRNAs targeting one or several regions of *XIST*. *Dots* indicate the mean for each biological replicate, n = 3 biological replicates with three technical replicates each. Data are normalized to the shMETTL3#1 sample and *ACTB*. *E*, SELECT assay for m^6^A levels at the crXIST#1-targeting site on *XIST*, when *METTL3* was knocked down, compared with cells with dCas13a-MTD and crRNAs targeting one or several regions of *XIST*. *Y*-axis indicates inverse normalized m^6^A abundance normalized to the shLUC sample. *Dots* indicate the mean for each biological replicate, n = 3 biological replicates with three technical replicates each. *F*, RT–qPCR of *GPC4* or *ATRX* when *METTL3* was knocked down, compared with cells cotransfected with dCas13a- FTO and crRNAs targeting one or several regions of *XIST*. *Dots* indicate the mean for each biological replicate, and the *bar* is the mean of all biological replicates, n = 3 biological replicates with three technical replicates each. Data are normalized to the shMETTL3#1 sample and *ACTB*. *G*, SELECT assay for m^6^A level at the crXIST#1-targeting site on *XIST*, when *METTL3* was knocked down, compared with shLUC transfected cells with dCas13a-FTO and crRNAs targeting one or several regions of *XIST*. *Y*-axis indicates inverse normalized m^6^A abundance normalized to the shLUC sample. *Dots* indicate the mean for each biological replicate, n = 3 biological replicates with three technical replicates each. crRNA, CRISPR RNA; m^6^A, *N*^6^-methyladenosine; qPCR, quantitative PCR; RIP-Seq, RNA immunoprecipitation sequencing; *XIST*, X-inactive specific transcript.

HEK293T cells were derived from a human embryonic kidney. However, prolonged *in vitro* cell culture has led to the presence of multiple X chromosomes (85), of which all are silenced except one active X (83). Consequently, they can serve as a model for X chromosome silencing (83). When we knocked down *METTL3* in 293T cells (Fig. 2, *E* and *F*), as previously reported, two X chromosome genes *GPC4* and *ATRX* were upregulated (Fig. 4, *A* and *B*) (7). *XIST* is marked by m^6^A on several sites of the linear RNA (Fig. 4*C*). Potentially, not all m^6^A on *XIST* is functional. Hence, we designed several crRNAs targeting three sites distributed along the length of the *XIST* transcript (crXIST#1–#3; Fig. 4*C*). We then cotransfected crRNAs targeting *XIST* with dCas13a-MTD in *METTL3* knockdown 293T cells to observe if the m^6^A methylation of *XIST* leads to suppression of X chromosome genes. RT–qPCR results showed that both *ATRX* and *GPC4* were significantly downregulated by dCas13a-MTD cotransfected with crRNAs targeting *XIST* (Fig. 4*D*). This suggests that m^6^A methylation of *XIST* RNA leads to suppression of these genes. SELECT assay using primers specific to the location of crXIST#1 showed significantly increased m^6^A methylation (Fig. 4*E*). Interestingly, in cells with crXIST#2 or #3, there was no significant change in the methylation at the site of crXIST#1 (Fig. 4*E*). This suggests that the range of dCas13a-MTD is relatively limited, and this contrasts with the effect seen in circular RNAs where the dCas13a-METTL3/MTD was effective at least 70 nucleotides away from the specific m^6^A measured in the SELECT assay (Fig. 1, *D* and *E*).

We next performed the same experiments but using dCas13a-FTO to detect X chromosome activation in WT cells. Transfection of the dCas13a-FTO/crXISTs into 293T cells led to a modest (but significant) upregulation of *GPC4* and *ATRX* expression (Fig. 4*F*). Presumably, demethylating *XIST* can only partially overcome epigenetic repression of the X chromosome. Interestingly, in an effect the same as dCas13a-MTD, the dCas13a-FTO/crXIST#1 combination led to demethylation of m^6^A specifically at the crXIST#1 site, but other crRNAs did not alter methylation at the crXIST#1 site (Fig. 4*G*). This indicates that on the linear *XIST* RNA, both dCas13a-MTD and dCas13a-FTO can modulate methylation over a limited range close to the crRNA targeting region. Overall, these results indicate that dCas13a-MTD and dCas13a-FTO can specifically alter m^6^A on *XIST* and lead to a change in the expression of X chromosome-associated genes.

### System for editing m^6^A on multiple RNAs

**Figure 5:**
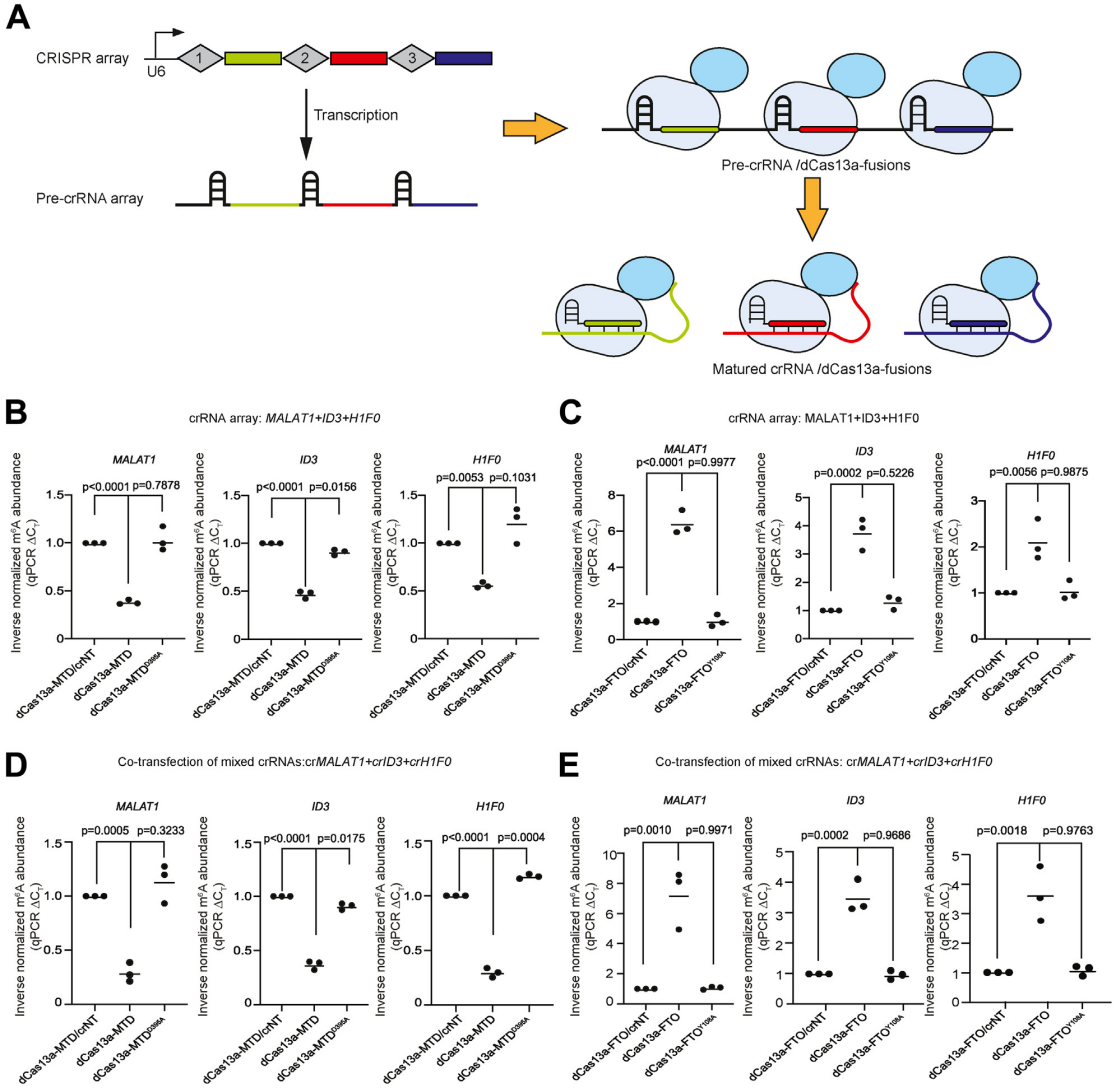
**dCas13a can process a multiple crRNA cassette for targeting multiple transcripts.***A*, schematic of a multiple crRNA cassette with crRNAs targeting three separate transcripts indicated by the *green*, *red*, and *blue boxes*. The CRISPR array is transcribed to form a pre-crRNA array, containing multiple crRNA scaffolds and the three crRNAs in serial. dCas13a fusions can then mature the pre-crRNAs inside the array to produce multiple mature crRNAs as the maturation domains of dCas13a are intact and functional. *B*, SELECT assay for inverse normalized m^6^A abundance of the three genes in the crRNA array, cotransfected with dCas13a-MTD or the catalytic-null dCas13a-MTD^D395A^, with the nontargeting crNT as a control, or with the crRNA array from *A*. m^6^A level is normalized to the crNT transfection. *Dots* indicate the mean for each biological replicate, and the *bar* is the mean of all biological replicates, n = 3 biological replicates with three technical replicates each. *C*, as in *B*, but using dCas13a-FTO and the catalytic-null dCas13a-FTO^Y108A^. *Dots* indicate the mean for each biological replicate, and the *bar* is the mean of all biological replicates, n = 3 biological replicates with three technical replicates each. *D*, as in *B*, individual vectors containing a single crRNA was used instead of the crRNA array. *Dots* indicate the mean for each biological replicate, and the *bar* is the mean of all biological replicates, n = 3 biological replicates with three technical replicates each. *E*, as in *B*, but using dCas13a-FTO and the catalytic-null dCas13a-FTO^Y108A^, and individual vectors containing a single crRNA was used instead of the crRNA array and using dCas13a-FTO and the catalytic-null dCas13a-FTO^Y108A^. *Dots* indicate the mean for each biological replicate, and the *bar* is the mean of all biological replicates, n = 3 biological replicates with three technical replicates each. crRNA, CRISPR RNA; m^6^A, *N*^6^-methyladenosine; MTD, methyltransferase domain.

As we have shown with the aforementioned *XIST* experiments, many RNAs have multiple enriched sites of m^6^A, and potentially several sites must be simultaneously targeted to achieve a biological effect. In addition, genes often have multiple transcript isoforms that can have divergent m^6^A patterns; hence, it would be useful to modulate m^6^A at several sites in a single transcript or multiple different genes “transcripts” simultaneously. In Cas13a, the two RNase domains and the pre-crRNA maturation domain are in two distant sites of Cas13a (48, 49). Hence, we reasoned that dCas13a is still capable of maturing a pre-crRNA array to multiple crRNAs. To investigate whether dCas13a can mature a pre-crRNA array to produce functional crRNAs, we constructed a crRNA array containing multiple crRNAs driven by a single U6 promoter (Fig. 5*A*). Coexpression of this vector in 293T cells with a crRNA array containing pre-crRNAs against *MALAT1*, *ID3*, and *H1F0* led to a significant increase in m^6^A level on all transcripts, as measured by SELECT assay, and the effect was not present if the catalytic-null dCas13a-MTD^D395A^ was used (Fig. 5*B*). dCas13a-FTO was also capable of processing the crRNA array and could simultaneously significantly decrease the level of m^6^A in all three transcripts (Fig. 5*C*). We also compared m^6^A editing efficiencies between the crRNA array and a cocktail of three single crRNAs. SELECT assay showed that the crRNA array was comparable to the efficiency achieved with mixed single crRNA vectors (Fig. 5, *D* and *E*). However, the multiple crRNA array has the advantage of a simplified transfection system, and a second advantage is that pooled crRNA vectors can transfect different cells randomly, whereas the crRNA array means that all crRNAs are present in the same cell. Whilst unlikely to be a problem in cell lines that have high transfection efficiency, in cell lines with poor transfection efficiency, it is helpful to guarantee that transfected cells will receive all crRNAs.

## Discussion

In this study, we have designed a CRISPR/dCas13a-associated system to edit the m^6^A methylation of circular RNA, mRNA, and lncRNAs. The catalytic-dead LwaCas13a (dCas13a) was fused to either the full-length m^6^A methyltransferase protein METTL3 (dCas13a-METTL3), the catalytical domain of METTL3 (dCas13a-MTD), or the demethylase protein FTO (dCas13a-FTO) to alter m^6^A modification status of targeted RNA sequences. We generated vectors suitable for liposomal or lentiviral transfection into cells. We validated the systems by exploring the impact of dCas13a fusions on m^6^A levels and the translation of GFP from an exogenous circular GFP reporter. The dCas13a fusions can also modulate m^6^A levels on linear endogenous transcripts, and these levels can impact transcript half-life. dCas13a is not perfect at targeting RNAs, and there is a possibility for off-target effects. We have demonstrated that the dCas13a fusions do not alter global levels of m^6^A, and overexpression did not affect endogenous levels of METTL3 or FTO. However, we cannot rule out other transcript-specific off-target effects. In the experiments reported here, the dCas13a fusions were expressed at around ∼20% of the endogenous levels of the corresponding proteins. It is possible the fusions work as dominant positives if expressed at higher levels or in cells with lower expression levels of the endogenous proteins.

Several research groups have demonstrated a role for m^6^A in modulating transcript half-life; however, a direct relationship between half-life and m^6^A has been challenging to demonstrate because of the widespread marking of transcripts with m^6^A, and its role in many biological processes. Here, we utilized our m^6^A editors to specifically alter the level of m^6^A on several RNAs. Using the dCas13a fusions, we manipulated the levels of m^6^A on specific transcripts, and this led to a change in transcript half-life. These experiments help establish a direct relationship between m^6^A levels and transcript half-life.

We also altered X chromosome activation status by editing m^6^A on *XIST*. This is a key result that demonstrates that m^6^A on *XIST* is crucial in enabling X chromosome suppression. Intriguingly, dCas13a-MTD could induce good suppression of X chromosome genes, suggesting m^6^A on *XIST* is required for initiation of X chromosome silencing. Knockdowns of *METTL3* can potently reactivate X chromosome genes (7); however, our dCas13a-FTO/cr*XIST* experiment led only to a modest, but significant, reactivation. Potentially, the loss of m^6^A on *XIST* has only a weak ability to overcome other X chromosome epigenetic suppression mechanisms. In addition, reductions in m^6^A on other transcripts may be required to achieve robust X chromosome reactivation. Previous studies exploring the role of m^6^A on *XIST* used whole-genome KOs or knockdowns of m^6^A-modulating enzymes, which will alter the m^6^A levels of several thousand transcripts that may indirectly modulate X chromosome inactivation. Here, we demonstrate that deposition of m^6^A on *XIST* alone is capable of silencing genes on the X chromosome, and removal of m^6^A on *XIST* can modestly reactivate X chromosome genes.

One interesting observation relates to the range of the dCas13a fusions and how far from the crRNA-binding site they can edit m^6^A. In the circular RNA, the m^6^A close to the ATG was altered even if the crRNA site was hundreds of nucleotides away. Conversely, in the linear *XIST* RNA experiments, all three crXISTs led to X gene activation or repression as appropriate; however, only when the crRNA was close to the m^6^A site did m^6^A levels change. This effect was the same for both dCas13a-METTL3 and dCas13a-FTO. This suggests that the dCas13a fusions have a limited range in linear RNAs but a longer range in circular RNAs. Potentially, this is due to the three-dimensional folding of the RNAs, and it will be interesting to see if this is a generalizable observation.

It is increasingly clear there is an intimate interdependence between epigenetic control of DNA and epitranscriptomic control of RNA. For example, m^6^A marks the majority of DNA:DNA:RNA R-loops in stem cells (86), and m^6^A has been identified as a critical regulator of chromatin structure, transregulation of gene expression, and stem cell differentiation (87, 88, 89). The dCas13a-fusion tools described here will be instrumental in dissecting these roles on specific individual transcripts and groups of transcripts. Utilizing this system, researchers can isolate the effects of m^6^A on individual transcripts to explore pluripotency, development, oncogenesis, and human disease.

## Experimental procedures

### dCas13a-METTL3, MTD, FTO plasmid construction

dCas13a fused with METTL3, MTD, and FTO separately, two nuclear localization signals, one hemagglutinin tag, and P2A mCherry are added inside each vector (Fig. S1*A*). A full list of Cas13 crRNAs used in this work is given in Table S1. Note that the D395A catalytic-null mutant on MTD is numbered according to the full-length METTL3. The plasmid backbones used for the lipofectamine-compatible system were pST1374 (Addgene: #13426) and pC0040-LwaCas13a crRNA backbone (Addgene: #103851). For the lentivirus system, dCas13a-M/F&crRNA vector, pFUGW/lentiCas9 (Addgene: #63592) was used. For the multiple crRNA vector, pC0040-LwaCas13a crRNA backbone (Addgene: #103851) was used.

### Cell culture and transfection

Cells were maintained at 37 °C with 5% CO_2_ in a humidified incubator and passaged every 2 to 3 days. WT HEK293T cells were cultured in high-glucose Dulbecco's modified Eagle's medium (Thermo Fisher Scientific) supplemented with 10% fetal bovine serum (Gibco). Cells were split with TrypLE Express (Life Technologies) according to the manufacturer’s instructions. HEK293T cells were seeded on 12-well poly (d-lysine) plates (Corning) in culture medium. At 80% confluency, approximately 12 h after plating, cells were transfected with 1250 ng of dCas13a m6A editor plasmid and 1250 ng of crRNA plasmid using 5 μl of Lipofectamine 3000 (Thermo Fisher Scientific) in Opti-MEM I Reduced Serum Media (Thermo Fisher Scientific). *Mettl3* KO mESCs were a kind gift from Jacob Hanna’s laboratory. The KO mESCs and matching WT mESC lines were cultured in fetal bovine serum–free N2B27-based media (90). About 500 ml of N2B27 media was generated by including 240 ml Dulbecco's modified Eagle's medium/F12 (Biological Industries—Hepes free, custom made), 240 ml neurobasal (Invitrogen; catalog no.: 21103), 5 ml N2 supplement (Invitrogen; catalog no.: 17502048 or in house prepared), 5 ml B27 supplement (Invitrogen; catalog no.: 17504044), 1 mM glutamine (Invitrogen), 1% nonessential amino acids (Invitrogen), 0.1 mM β-mercaptoethanol (Sigma), and penicillin–streptomycin (Invitrogen). Naïve conditions for mESC included 10 μg recombinant human LIF (Peprotech) and small-molecule inhibitors CHIR99021 (CH, 1–3 μM; Axon Medchem) and PD0325901 (PD, 1 μM; TOCRIS) termed 2i. mESCs were cultured on fibronectin-coated plates where indicated. Naïve pluripotent cultures were passaged following 0.25% trypsinization every 3 to 4 days.

### Lentivirus production

To produce lentivirus, HEK293T cells were transiently transfected with lentivirus constructs and cotransfected with the packaging plasmids pCMV-dR8.91 and pMD2.G. Lentivirus was collected 48 h after transfection and filtered through 0.45 μm filters. The virus supernatant was centrifuged at 1500*g* for 30 min at 4 °C to collect pellets. The pellets were resuspended in cold culture medium and directly added to cells or frozen at −80 °C. The resulting lentiviral particles were used to generate mESCs stably expressing Cas13a m^6^A editors. The mESCs were transduced with lentivirus pU6-crRNA-EF1a-dCas13a-METL3/FTO-2A-mCherry.

### RNA isolation and RT–qPCR

Total RNA was isolated from WT or transiently transfected cells with MiniBEST Universal RNA Extraction Kit (Takara), an additional DNase I (NEB) digestion step was performed on all samples to avoid DNA contamination, and RNA concentration was measured by Nanodrop (Thermo Fisher Scientific). Total RNA was extracted using MiniBEST Universal RNA Extraction Kit. First-strand cDNA was synthesized by reverse transcription of 1 μg RNA using PrimeScript RT Master Mix (Takara). Quantitative real-time PCR was performed using TB Green Premix Ex Taq (Takara) in QuantStudio 7 Flex Real-Time PCR System (Life Technologies). βACTIN and GAPDH were used as reference genes for input normalization. The mRNA expression was measured by qPCR using the ΔΔCT method. Primers for qPCR are listed in Table S1.

### METTL3 knockdown *via* shRNA

The shRNA targeting *METTL3* used in this study was previously described (9, 20) and was cloned into an shRNA expression backbone vector pSH. At 80% confluency, approximately 12 h after plating, cells were transfected with 2500 ng of shMETTL3 plasmid and 2500 ng of shControl (luciferase) plasmid separately with 5 μl of Lipofectamine 3000 in Opti-MEM I Reduced Serum Media. Cells were maintained at 70 to 80% confluency and collected 48 h after the transfection. Knockdown was confirmed by RT–qPCR and Western blot.

### RNA half-life assay

HEK293T cells and mESCs for lifetime assay were cultured in 12-well plates, cultured cells were transfected with the dCas13a fusions including different crRNAs, and the control crRNA separately at 50% confluency. After 12 h, each well of a 12-well plate was reseeded into three wells in a 12-well plate, and each well was controlled to contain the same number of cells. After 48 h, actinomycin D was added at a concentration of 5 μg/ml at 4, 2, and 0 h before total RNA was extracted by MiniBEST Universal RNA Extraction Kit. The abundances of the target genes were measured at each time point by RT–qPCR using GAPDH as a reference gene. The degradation rate of RNA, *k*, was calculated by:

$${\log\;}_{2}\left(\frac{At}{A0}\right)=-kt$$

where *t* is transcription inhibition time (h), *At* and *A*0 represent mRNA quantity (attomole) at time *t* and time 0. Two *k* values were calculated: time 2 h *versus* time 0 h and time 4 h *versus* time 0 h. The final half-life was calculated by using the average of *k* 2 h and *k* 4 h:

$$t^{\frac{1}{2}}=\frac{kt2\text{h}+kt4\text{h}}{2}$$

### SELECT assay

The SELECT assay was performed as previously described (76). Briefly, 80 fmol synthesized RNA oligo was mixed with 40 nM Up-T Primer and 40 nM Down Primer in 18 μl 1× reaction buffer. 1× CutSmart buffer (50 mM KAc (Ac=CH_3_COO), 20 mM Tris–HAc, 10 mM MgAc_2_, 100 μg/ml bovine serum albumin, pH 7.9 @ 25 °C) was used to examine SplintR ligase, T4 DNA ligase, and T4 RNA ligase 2 (dsRNA ligase). 1× T3 DNA ligase reaction buffer (66 mM Tris–HCl, 10 mM MgCl_2_, 1 mM ATP, 1 mM DTT, 7.5% PEG 6000, pH 7.6 @ 25 °C) was used with T3 DNA ligase and T7 DNA ligase. 1× 9°N DNA ligase reaction buffer (10 mM Tris–HCl, 600 μM ATP, 2.5 mM MgCl_2_, 2.5 mM DTT, 0.1% Triton X-100, pH 7.5 @ 25 °C) was used. 1× Taq DNA ligase reaction buffer (20 mM Tris–HCl, 25 mM KAc, 10 mM MgAc_2_, 10 mM DTT, 1 mM NAD, 0.1% Triton X-100, pH 7.6 @ 25 °C) was used in the test of Taq DNA ligase. The RNA and primers were annealed by incubating the mixture at a temperature gradient: 90 °C for 1 min, 80 °C for 1 min, 70 °C for 1 min, 60 °C for 1 min, 50 °C for 1 min, and then 40 °C for 6 min. A 2 μl mixture containing ligase with indicated concentration and 10 nmol ATP (only added in the test of SplintR ligase, T4 DNA ligase, and T4 RNA ligase 2) was added to the former annealed mixture. The final reaction mixture was incubated at 37 °C for 20 min and then denatured at 95 °C for 5 min and kept at 4 °C. Subsequently, RT–qPCR was carried out as described previously. SELECT primers are listed in Table S1.

### Western blot

Whole-cell extracts were extracted by directly lysing the cells with 1× radioimmunoprecipitation assay buffer (Beyotime) with 1 mM PMSF (Beyotime) added immediately before use. Samples were boiled by adding 6× SDS sample buffer for 10 min at 100 °C and resolved using SDS-polyacrylamide gel electrophoresis. The proteins were probed with the following antibodies: monoclonal anti-GFP (1:2000 dilution; Thermo Fisher Scientific), anti-βACTIN (1:2000 dilution; Thermo Fisher Scientific), and anti-METTL3 (1:1000 dilution; Abcam), and anti-FTO (1:2000 dilution; Abcam). Immunodetection was performed using horseradish peroxidase–conjugated Affinipure goat anti-mouse IgG (H + L) (1:5000 dilution; catalog no.: SA0 001-1; Proteintech) or horseradish peroxidase–conjugated Affinipure goat anti-Rabbit IgG (H + L) (1:5000 dilution; catalog no.: SA00001-2; Proteintech) and ECL prime substrate (Bio-Rad) according to the manufacturer's instructions.

### Whole transcriptome m^6^A measurements

Global m^6^A/m in total RNA was quantified by the EpiQuik m^6^A/m RNA Methylation Quantification Kit (Epigentek Group) following the manufacturers’ specifications and using 100 ng as input.

### m^6^A MeRIP–qPCR

MeRIP–qPCR was performed using an EpiMark N6-Methyladenosine Enrichment Kit (catalog no. E1610S; New England Biolabs), according to the manufacturer’s instructions with some modifications. Briefly, total RNA was fragmented in a solution of 50 mM Tris–HCl, pH 8.0, 50 mM MgCl_2_, and heated at 95 °C for 8 min. The m^6^A-modified and m^6^A-unmodified control RNAs were spiked into the fragmented RNA, and a portion was saved as input RNA. The remaining fragmented RNA was subjected to m^6^A IP: 30 μl of protein G magnetic beads were washed twice by IP reaction buffer (150 mM NaCl, 10 mM Tris–HCl, pH 7.5, 0.1% NP-40 in nuclease-free water), resuspended in 250 μl of reaction buffer, and tumbled with 5 μg of anti-m^6^A antibody at 4 °C overnight. After two washes in reaction buffer, the antibody–bead mixture was resuspended in 500 μl of the reaction mixture containing 10 μg of fragmented total RNA, 100 μl of reaction buffer, and 5 μl of RNasin Plus RNase Inhibitor (Promega), and incubated for at least 4 h at 4 °C. To remove unbound RNA, samples were washed 5× with each of the following buffers: reaction buffer (150 mM NaCl, 10 mM Tris–HCl, pH 7.5, 0.1% NP-40 in nuclease-free water), low-salt reaction buffer (50 mM NaCl, 10 mM Tris–HCl, pH 7.5, 0.1% NP-40 in nuclease-free water), and high-salt reaction buffer (500 mM NaCl, 10 mM Tris–HCl, pH 7.5, 0.1% NP-40 in nuclease-free water). RNA was eluted in RLT buffer (Qiagen) and purified with RNA Clean & Concentrator-5 kits (Zymo Research). Purified RNA was reverse transcribed with High-Capacity RNA-to-cDNA (Thermo Fisher Scientific) according to the manufacturer’s protocol. The resulting cDNA was preamplified with SsoAdvanced PreAmp Supermix (Bio-Rad) according to the manufacturer’s protocol. qPCR was performed with IQ Multiplex Powermix (Bio-Rad). All reactions were performed and quantified on a CFX96 Real-Time PCR Detection System (Bio-Rad). MeRIP–qPCR primers are listed in Table S1.

### Fluorescent-activated cell sorting–based analysis of cell cycle and cell death

Cell cycle and cell death were measured using Cell Cycle and Apoptosis Detection Kit (Beyotime; catalog no.: 1052), according to the manufacturer’s instructions. Briefly, transfected 293T cells were grown on a well in a 6-well plate for 36 h. Cells were washed with cold PBS, fixed in 70% ethanol, and stored at 4 °C for subsequent cell cycle analysis. Fixed cells were washed with PBS once and then resuspended in 1 ml of propidium iodide staining reagent. Samples were incubated in the dark for 30 min before cell cycle analysis. The distribution of cells in the cell cycle was measured by flow cytometer (BD FACSCalibur), and quantitation of cell cycle distribution was performed using Multi-cycle Software (ModFit software). The percentage of cells in the G1, S, and G2 phases and cell death was calculated.

### Statistical procedures

Significance was calculated from a two-tailed unpaired Student’s *t* test for all indicated figure panels with a *p* value except for Figures 1, *F* and *G* and S5, *E* and *F*. Significance tests were calculated using GraphPad Prism 8 (GraphPad Software, Inc). Biological replicates were defined as experiments performed using different cells on different days. Technical replicates were defined as multiple repeats of the same biological replicate in PCR-based assays (SELECT, MeRIP–qPCR, and RT–qPCR).

## Data availability

All data are contained within the article. Plasmids will be available upon request.

## Supporting information

This article contains supporting information (4, 91).

## Conflict of interest

The authors declare that they have no conflicts of interest with the contents of this article.
