# Supplementary material for: A programmable system to methylate and demethylate N6-methyladenosine (m6A) on specific RNA transcripts in mammalian cells
Source: J Biol Chem. 2022 Sep 23;298(11):102525. doi: 10.1016/j.jbc.2022.102525 (PMC9597892; doi:10.1016/j.jbc.2022.102525)
Supplement: Supplementary data [file mmc1.pdf]

A programmable system to methylate and demethylate m^{6}A on specific RNA transcripts in mammalian cells

Chen Chang[1,2], Gang Ma[1,3], Edwin Cheung[2]*, Andrew P. Hutchins[1,3]*

[1]Department of Biology, School of Life Sciences, Southern University of Science and Technology, Shenzhen, China

[2]Cancer Centre, Faculty of Health Sciences, University of Macau, Taipa, Macau SAR

[3]Shenzhen Key Laboratory of Gene Regulation and Systems Biology, School of Life Sciences, Southern University of Science and Technology, Shenzhen, China

*Correspondence: andrewh@sustech.edu.cn; echeung@um.edu.mo

Supplementary Figures

Supplementary Figure 1. Transfection of the dCas13a-fusions into 293T cells.

Supplementary Figure 2. Circular RNA m^{6}A reporter structure.

Supplementary Figure 3. Transfection of dCas13a-fusion proteins does not impact the levels of endogenous METTL3 or FTO.

Supplementary Figure 4. Genome views of m^{6}A levels of transcripts targeted by the crRNAs in 293T cells.

Supplementary Figure 5. m^{6}A editors can methylate and demethylate endogenous mRNA and long non-coding RNA transcripts.

Supplementary Figure 6. Lentiviral vectors for editing m^{6}A in mESCs.

References

1. Wang, Y., Xiao, Y., Dong, S., Yu, Q., and Jia, G. (2020) Antibody-free enzyme-assisted chemical approach for detection of N6-methyladenosine. *Nature Chemical Biology*

2. Meyer, K. D., Saletore, Y., Zumbo, P., Elemento, O., Mason, C. E., and Jaffrey, S. R. (2012) Comprehensive analysis of mRNA methylation reveals enrichment in 3' UTRs and near stop codons. *Cell* **149**, 1635-1646

3. Batista, P. J., Molinie, B., Wang, J., Qu, K., Zhang, J., Li, L., Bouley, D. M., Lujan, E., Haddad, B., Daneshvar, K., Carter, A. C., Flynn, R. A., Zhou, C., Lim, K. S., Dedon, P., Wernig, M., Mullen, A. C., Xing, Y., Giallourakis, C. C., and Chang, H. Y. (2014) m(6)A RNA modification controls cell fate transition in mammalian embryonic stem cells. *Cell Stem Cell* **15**, 707-719

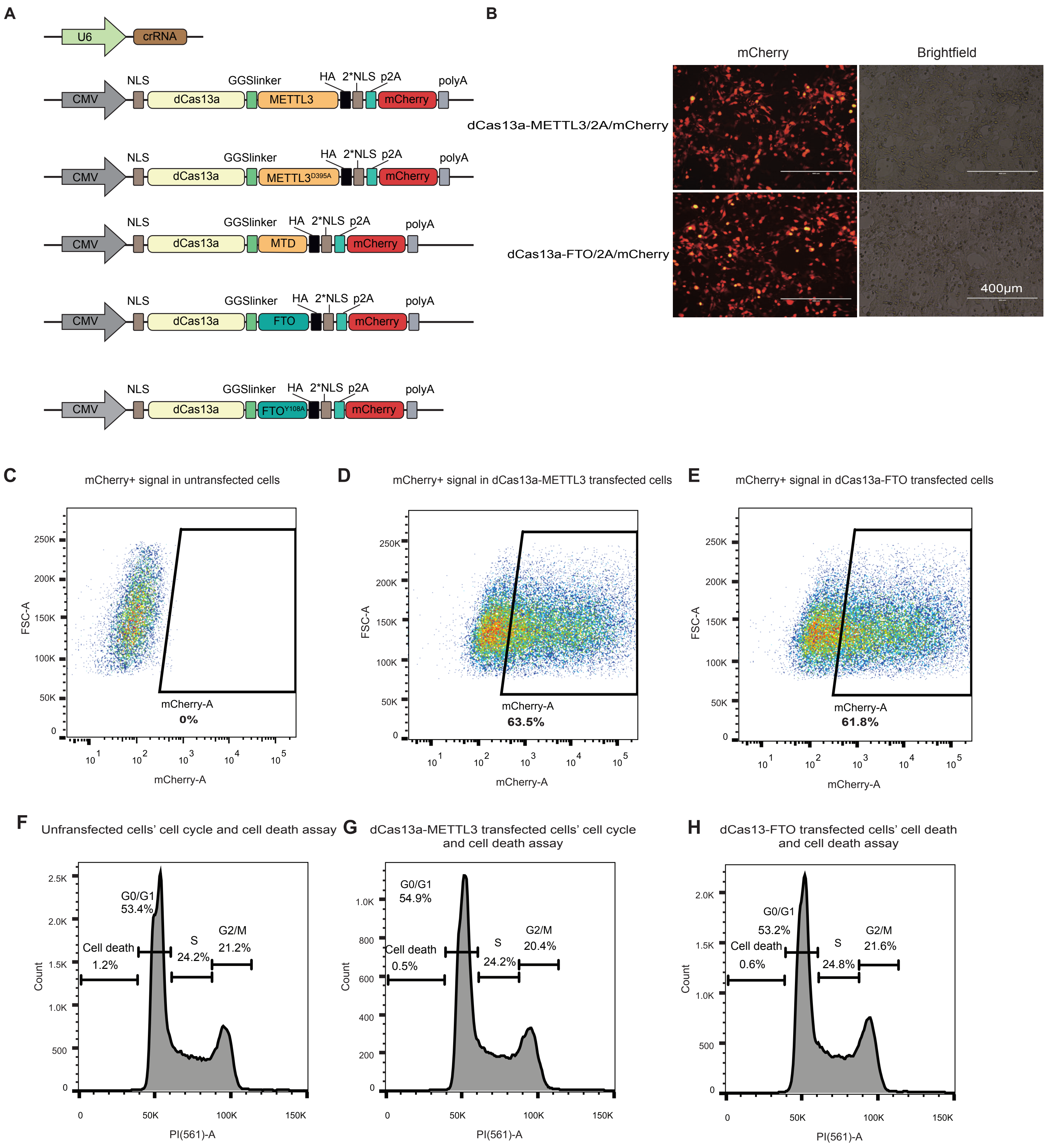

Supplementary Figure 1

Supplementary Figure 1. Transfection of the dCas13a-fusions into 293T cells.

A. Schematic of the constructs made in this manuscript suitable for lipid or electroporation-based transfection. NLS=nuclear localization signal; HA=hemagglutinin tag; p2A=self-cleaving peptide sequence.
B. mCherry transfection images of the dCas13a-METTL3 or dCas13a-FTO constructs in 293T cells.
C. FACS analysis showing forward scatter (FSC-A) versus mCherry fluorescence, for untransfected cells.
D. As in panel C, but for dCas13a-METTL3 and crNT transfected cells.
E. As in panel E, but for dCas13a-FTO and crNT transfected cells.
F. FACS cell cycle and cell death analysis for untransfected cells.
G. As in panel F, but with dCas13a-METTL3/crNT transfection.
H. As in panel F, but with dCas13a-FTO/crNT transfection.

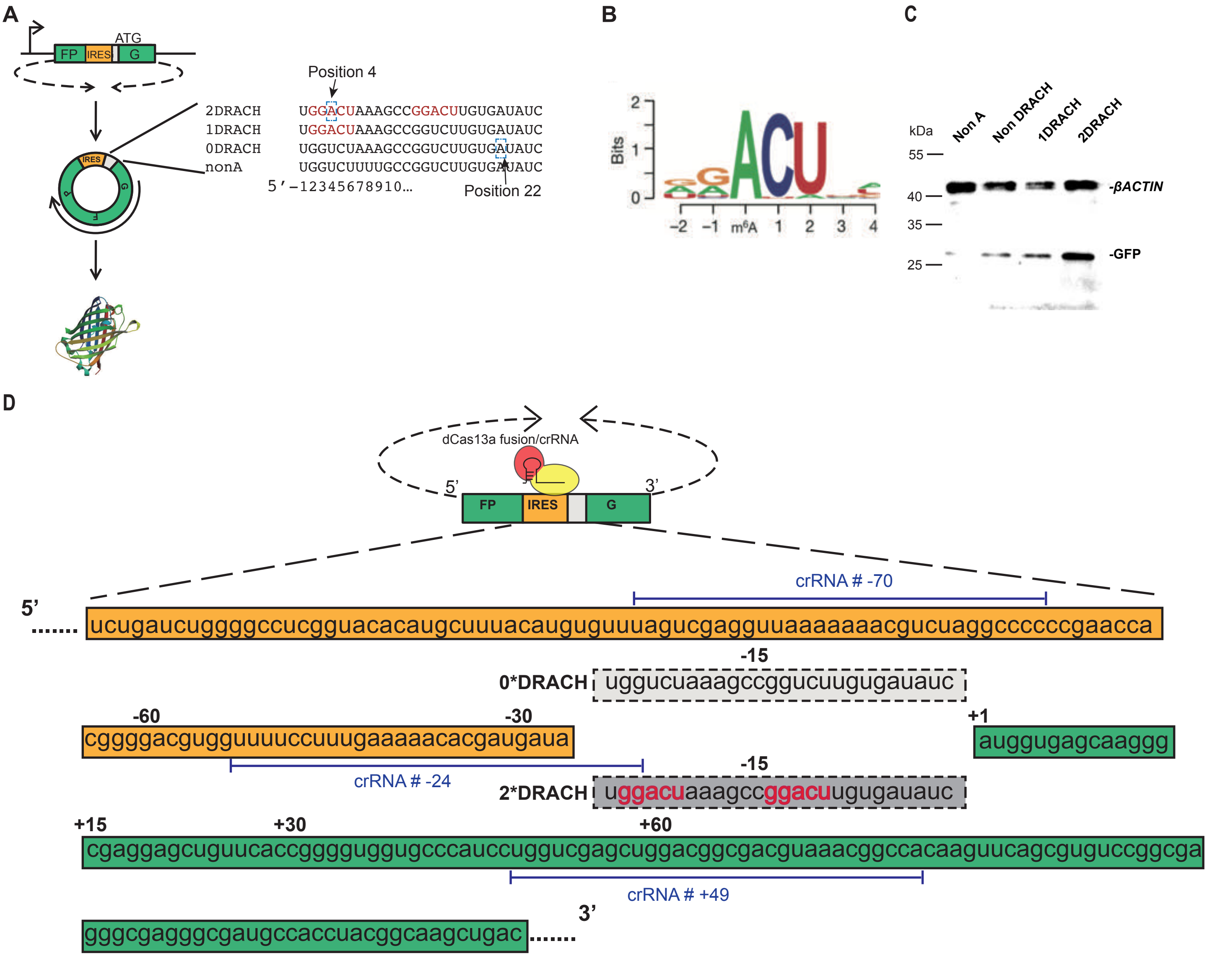

Supplementary Figure 2

Supplementary Figure 2. Circular RNA m^{6}A reporter structure.

A. Schematic of the circRNA construct. Sequences of the DRACH are indicated and the positions of specific A nucleotides used in the SELECT assay.

B. DRACH m^{6}A position weight matrix motif.

C. Western blot of GFP translated from circular RNA constructs containing 2, 1 or 0 DRACH motifs or a sequence with no A nucleotides. This experiment was performed once. Molecular weight markers (in kilo Daltons; kDa) are indicated on the right side for this and all subsequent Western blots.

D. Schematic of the zoomed in region between the IRES (in orange) and the ATG (at position +1) of GFP. The sequences of the 0DRACH and 2DRACH constructs are shown. Locations of the crRNAs are relative to the indicated nucleotide position numbers, numbered according to the location of the ATG (at nucleotide 1).

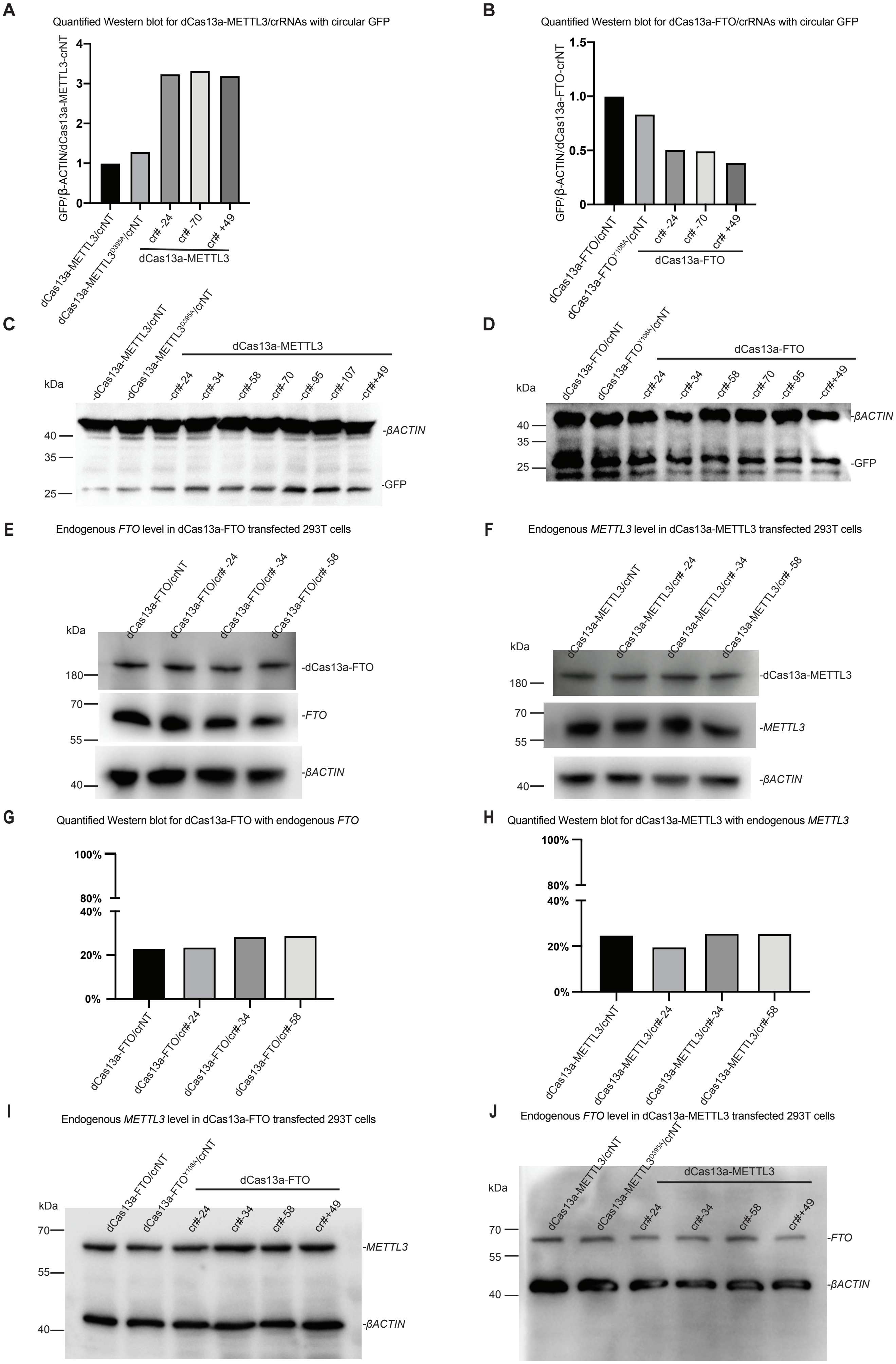

Supplementary Figure 3

Supplementary Figure 3. Transfection of dCas13a-fusion proteins does not impact the levels of endogenous METTL3 or FTO.

A. Quantification of the Western blot result in **Figure 1B**. Quantitation is normalized to dCas13a-METTL3/crNT.
B. Quantification of the Western blot result in **Figure 1C**. Quantitation is normalized to dCas13a-FTO/crNT.
C. Western blot of GFP from 293T cells transfected with crNT, or crRNAs targeting specific sites around the ATG codon of the circular plasmid, and dCas13a-METTL3, or the catalytic dead dCas13a-METTL3^{D395A}. This experiment was repeated 3 times with similar results. Molecular weight markers (in kDa) are indicated on the left side for this and all subsequent Western blots.
D. Western blot of GFP from 293T cells transfected with crNT, or crRNAs targeting specific sites around the ATG codon of the circular plasmid, and dCas13a-FTO, or the catalytic dead dCas13a-FTO^{Y180A}. This experiment was repeated 3 times with similar results.
E. Western blot of endogenous FTO protein, dCas13a-FTO fusion and βACTIN (control) in 293T cells transfected with the indicated dCas13a-FTO fusions and various crRNAs.
F. Western blot of endogenous METTL3 protein, dCas13a-METTL3 fusion and beta-ACTIN (control) in 293T cells transfected with the indicated dCas13a-METTL3 fusions and various crRNAs.
G. Quantification of dCas13a-FTO from the Western blot result in **panel E**. Quantitation is normalized to endogenous FTO.
H. Quantification of dCas13a-METTL3 from the Western blot result in **panel F**. Quantitation is normalized to endogenous FTO.
I. Western blot of endogenous METTL3 protein and βACTIN (control) in 293T cells transfected with the indicated dCas13a-FTO fusions and various crRNAs.
J. Western blot of endogenous FTO protein and βACTIN (control) in 293T cells transfected with the indicated dCas13a-METTL3 fusions and various crRNAs.

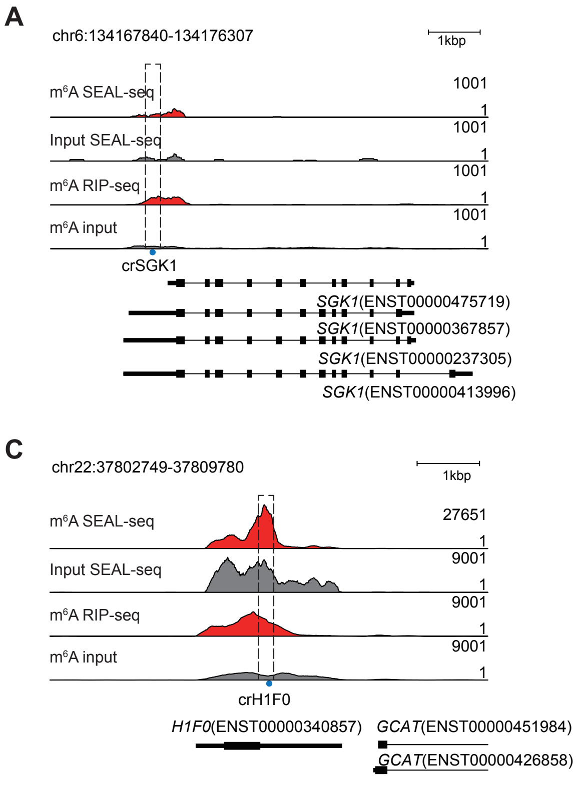

Supplementary Figure 4

Supplementary Figure 4. Genome views of m^{6}A levels of transcripts targeted by the crRNAs in 293T cells.

A. Genome view (hg38 genome assembly) of m^{6}A RIP-seq data in 293T cells at the *SGK1* locus. The location of the crRNA is indicated with a dotted box. Red indicates m^{6}A enrichment data, grey tracks indicate the corresponding input data. Transcripts are from GENCODE v32. m^{6}A abundance data is from GSE129979 (1) (top two rows) or GSE29714 (2) (bottom two rows).

B. As in **panel A**, but showing *MALAT1*.

C. As in **panel A**, but showing *H1F0*.

D. As in **panel A**, but showing *ID3*.

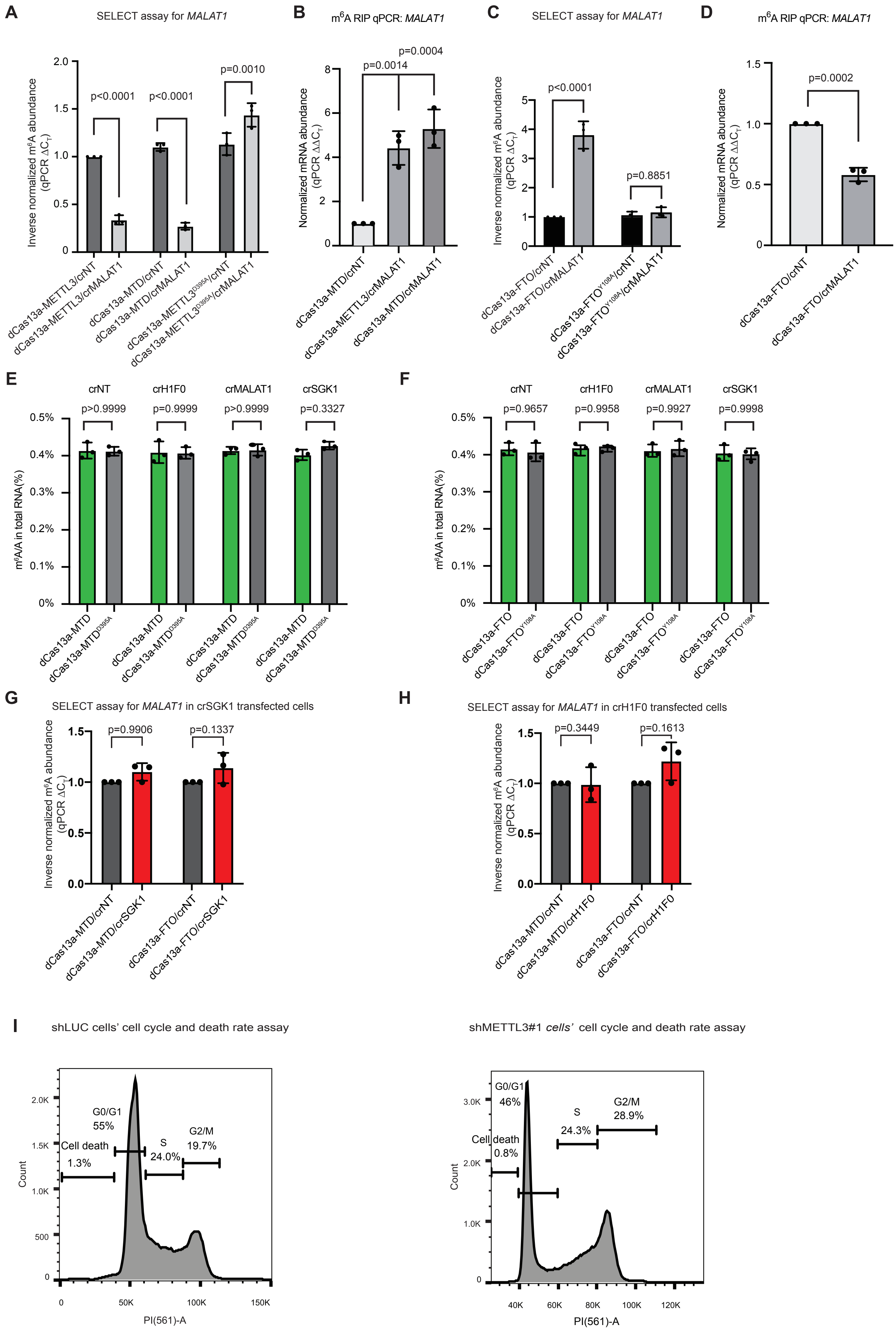

Supplementary Figure 5

Supplementary Figure 5. m^{6}A editors can methylate and demethylate endogenous mRNA and long non-coding RNA transcripts.

A. SELECT assay for *MALAT1*, with dCas13a-MTD, dCas13a-METTL3 or the catalytic null, with a non-targeting crRNA or with a crRNA targeting *MALAT1*. Y axis indicates inverse normalized m^{6}A abundance normalized to dCas13a-METTL3/crNT sample. Dots indicate the mean for each biological replicate and the bar is the mean of all biological replicates, n=3 biological replicate with 3 technical replicates each.

B. MeRIP-qPCR for *MALAT1* transcript, in cells transfected with dCas13a-METTL3, or dCas13a-MTD and non-targeting crRNA, or a crRNA targeting *MALAT1*. Dots indicate the mean for each biological replicate and the bar is the mean of all biological replicates, n=3 biological replicate with 3 technical replicates each. Data is normalized to dCas13a-METTL3/crNT sample and against the input.

C. SELECT assay for *MALAT1*, with dCas13a-FTO or the catalytic null form, with a non-targeting crRNA or with a crRNA targeting *MALAT1*. Y axis indicates inverse normalized m^{6}A abundance normalized to dCas13a-FTO/crNT sample. Dots indicate the mean for each biological replicate and the bar is the mean of all biological replicates, as n=3 biological replicate with 3 technical replicates each.

D. MeRIP-qPCR for *MALAT1* transcript, in cells transfected with dCas13a- FTO, or dCas13a-MTD and non-targeting crRNA (crNT), or a crRNA targeting *MALAT1*. Dots indicate the mean for each biological replicate and the bar is the mean of all biological replicates, n=3 biological replicate with 3 technical replicates each.

E. ELISA for ratio of m^{6}A/A in the indicated cells transfected with dCas13a-MTD, or its catalytic null, with the indicated crRNAs. Dots indicate the mean for each biological replicate and the bar is the mean of all biological replicates, n=3 biological replicates with 3 technical replicates each. p-value is from a one-way ANOVA.

F. As in **panel E**, but using the dCas13a-FTO and its catalytic null.

G. SELECT assay for *MALAT1* in crSGK1 transfected cells. Y axis indicates inverse normalized m^{6}A abundance normalized to dCas13a-METTL3 or dCas13a-FTO/crNT sample. Dots indicate the mean for each biological replicate and the bar is the mean of all biological replicates, n=3 biological replicate with 3 technical replicates each.

H. SELECT assay for *MALAT1* in crH1FO transfected cells. Y axis indicates inverse normalized m^{6}A abundance normalized to dCas13a-METTL3 or dCas13a-FTO/crNT sample. Dots indicate the mean for each biological replicate and the bar is the mean of all biological replicates, n=3 biological replicate with 3 technical replicates each.

I. FACS cell cycle and cell death analysis for untransfected cells or cells transfected with an shRNA against *METTL3*.

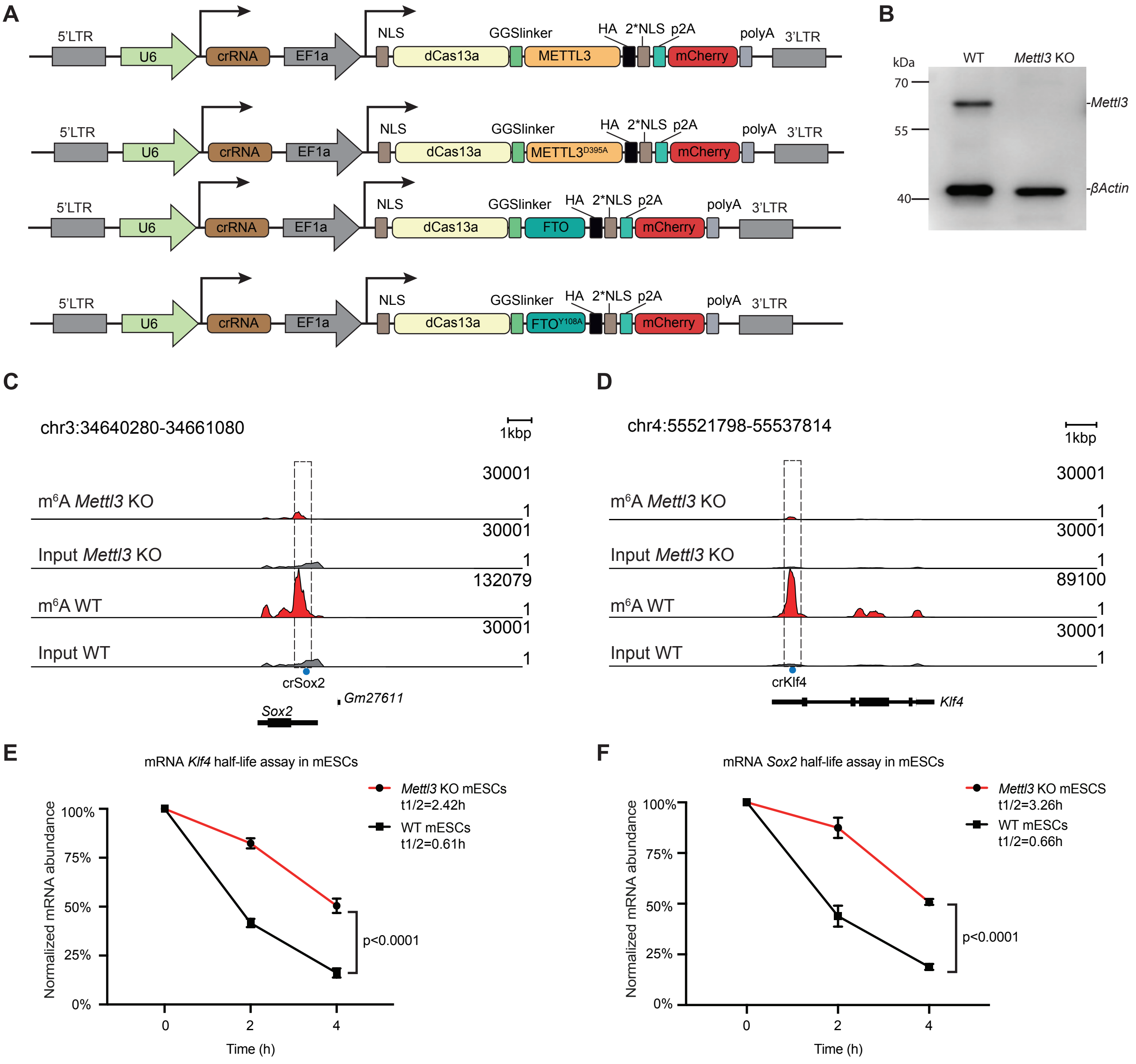

Supplementary Figure 6

Supplementary Figure 6. Lentiviral vectors for editing m^{6}A in mESCs.

A. Schematic of the lentiviral vectors generated in this study. NLS=nuclear localization signal; HA=hemagglutinin tag; p2A= self-cleaving peptide sequence; LTR=long terminal repeat.

B. Western blot of METTL3 in *Mettl3* knockout (KO) mESCs and wildtype (WT) mESCs.

C. Genome view (mm10) of m^{6}A RIP-seq data in mESCs cells at the *Sox2* loci. The location of the crRNA is indicated with a blue dot. Red indicates m^{6}A enrichment data, grey indicates the corresponding input data. Data represents m^{6}A RIP-seq from *Mettl3* KO and WT cells. Transcripts are from GENCODE M.v25. m^{6}A abundance data is from GSE52662 (3).

D. As in **panel B**, but for the *Klf4* locus.

E. Half-life comparison of *Klf4* between *Mettl3* KO mESCs and WT mESCs. Data is normalized to their respective 0-hour time points.

F. Half-life comparison of *Sox2* between *Mettl3* KO mESCs and WT mESCs. Data is normalized to their respective 0-hour time points.
